# Psychometric properties and outcomes of the PROMIS Sexual Function and Satisfaction measures in individuals with congenital bleeding disorders

**DOI:** 10.1016/j.rpth.2026.106937

**Published:** 2026-09-02

**Authors:** Tessa C.M. van Gastel, Lorynn Teela, Michiel A.J. Luijten, Vicky Lehmann, Marjolein Peters, Caroline B. Terwee, Michiel Coppens, Marja Voskuilen, Saskia E.M. Schols, Paula F. Ypma, A.J. Gerard Jansen, Paul L. den Exter, Laurens Nieuwenhuizen, Floor C.J.I. Heubel-Moenen, Karin P.M. van Galen, Karin Fijnvandraat, Lotte Haverman

**Affiliations:** 1 Amsterdam UMC Location University of Amsterdam, Emma Children’s Hospital, Child and Adolescent Psychiatry & Psychosocial Care, Amsterdam, The Netherlands; 2 Amsterdam Reproduction and Development, Child Development, Amsterdam, The Netherlands; 3 Amsterdam Public Health, Mental Health and Health Behaviors & Chronic Diseases, Amsterdam, The Netherlands; 4 Amsterdam Public Health, Mental Health and Digital Health, Amsterdam, The Netherlands; 5 Amsterdam Public Health, Mental Health and Methodology, Amsterdam, The Netherlands; 6 Department of Medical Psychology, Amsterdam University Medical Center, University of Amsterdam, Amsterdam, The Netherlands; 7 Amsterdam UMC Location University of Amsterdam, Emma Children’s Hospital, Paediatric Haematology, Amsterdam, The Netherlands; 8 Department of Epidemiology and Data Science, Amsterdam UMC, Vrije Universiteit Amsterdam, The Netherlands; 9 Amsterdam Public Health Research Institute, Methodology and Quality of Care, Amsterdam, The Netherlands; 10 Amsterdam Cardiovascular Sciences, Department of Vascular Medicine, Amsterdam, The Netherlands; 11 Department of Hematology, University Medical Center Groningen, Groningen, The Netherlands; 12 Department of Hematology, Radboud university medical center, Nijmegen, The Netherlands; 13 Department of Hematology, Haga Hospital, The Hague, The Netherlands; 14 Department of Hematology, Erasmus MC, University Medical Center, Rotterdam, The Netherlands; 15 Department of Medicine, Thrombosis and Hemostasis, Leiden University Medical Center, Leiden, The Netherlands; 16 Department of Internal Medicine, Maxima Medical Center, Eindhoven, The Netherlands; 17 Department of Internal Medicine/Hematology, Maastricht University Medical Center+, Hemophilia Treatment Center Nijmegen-Eindhoven-Maastricht, Maastricht, The Netherlands; 18 Cardiovascular Research Institute Maastricht (CARIM), Maastricht, The Netherlands; 19 Van Creveldkliniek, University Medical Center Utrecht, Utrecht University, Utrecht, The Netherlands

**Keywords:** hemophilia, patient-reported outcome measures (PROMs), psychometric properties, reliability, sexual function, sexual satisfaction, validity, von Willebrand disease

## Abstract

**Background:**

Sexual function can be negatively affected in individuals with congenital bleeding disorders (CBDs). Patient-reported outcome measures can provide insight into patients’ experiences and help address sexual function and sexual satisfaction in clinical care.

**Objectives:**

This study evaluates the measurement properties of a selection of PROMIS Sexual Function and Satisfaction (PROMIS SexFS) measures in adults with CBDs and describes the outcomes.

**Methods:**

In this cross-sectional multicenter study, men with hemophilia and women with hemophilia or von Willebrand disease were invited to participate. Six screener items, 12 measures from the PROMIS SexFS, and disease-specific items on sexual function and satisfaction were completed. Reliability was evaluated using standard error of T-scores. Internal consistency was evaluated using item–total correlation. Known-group validity was examined by testing predefined hypotheses. Feasibility was evaluated using percentage of lowest and highest scores, and relevance was evaluated qualitatively. Lastly, PROMIS T-scores were compared with reference data from the Dutch general population using independent samples *t*-test.

**Results:**

Data from 231 patients (mean age 44 years, range 18-81 years; 54% male) were collected. For all measures, >78% met the reliability criterion. Internal consistency and feasibility were sufficient, and known-group validity was supported. Compared with reference data, patients reported greater orgasm pleasure (d = 0.23) and more interest in sexual activity (d = 0.19). Males reported better erectile function (d = 0.26), while females reported more vaginal discomfort with sexual activity (d = 0.48).

**Conclusion:**

The Dutch PROMIS SexFS measures showed sufficient reliability and validity for assessing sexual function and satisfaction in individuals with CBDs. It can inform research and support clinical discussions.

## Essentials

• This study investigates selected PROMIS Sexual Function and Satisfaction (PROMIS SexFS) measures.
• This is a cross-sectional multicenter study in 231 adults with a congenital bleeding disorder.
• The PROMIS SexFS is sufficiently valid and reliable to assess sexual function and satisfaction.
• Insights from the PROMIS SexFS can inform research and support clinical discussions.

## 1 Introduction

Despite advances in treatment options [[1], [2], [3]], living with a congenital bleeding disorder (CBD) such as hemophilia or von Willebrand disease (VWD) can have a significant impact on daily life [4]. Individuals with CBDs may experience limitations in joint movement in case of recurrent bleeds, excessive bleeding after trauma or surgery, easy bruising, and pain [[5], [6], [7]]. Women with CBDs can also experience heavy and prolonged menstrual bleeding [8], which can interfere with physical function, social participation, and work/education [4,9,10].

Sexual function and sexual satisfaction are important areas of daily life that can also be affected by CBDs, but they are often neglected in clinical care. Our research group [11] and previous research has shown that individuals with CBDs may feel limited in their sexual activity due to fear of bleeds, the need to be cautious during intimacy, and mental (eg, lower self-esteem) and physical (eg, limited joint movement) complaints [[12], [13], [14], [15], [16]]. Specifically for women with CBDs, concerns about blood loss, lack of sex drive, prolonged menstruation, or partners’ discomfort with bleeding can negatively affect their sex life and satisfaction [12]. Men with hemophilia may experience hematospermia, leading to insecurities about sexual activities [11,17,18]. Despite these challenges, individuals with CBDs report a lack of available information and limited discussion of sexual function and sexual satisfaction in clinical care [12,13,15,17,19].

An approach to gain insights into sexual function and sexual satisfaction and to facilitate conversations with clinicians is to use patient-reported outcome measures (PROMs) [20,21]. PROMs are standardized questionnaires that are completed by patients themselves, to assess their perspective on various domains of health (patient-reported outcomes) [22]. Previously developed PROMs assessing sexual function and sexual satisfaction differ in quality and depth [21], as many focus primarily on the physical domains of sexual function and neglect sexual satisfaction [16,23]. Additionally, research sometimes relies on single, binary items or nonvalidated items to assess sexual function [17,24,25]. Consequently, the PROMIS Sexual Function and Satisfaction (PROMIS SexFS) is a promising set of measures [26] that can be used to assess over 10 different domains of sexual function and sexual satisfaction, such as vaginal discomfort, erectile function, and factors interfering with sexual satisfaction [21]. The PROMIS SexFS v2.0 was developed in the United States, and 12 measures and the Sexual Function Screener items were translated and validated for use in the Netherlands [27]. There is growing evidence supporting the validity of generic PROMIS measures across patient populations [[28], [29], [30], [31]], and further validation of these measures may therefore not be necessary. However, the performance of the PROMIS SexFS measures in adults with CBDs has not yet been evaluated. Therefore, this study aimed to assess measurement properties, feasibility, and relevance of the 12 translated PROMIS SexFS measures in adults with CBDs and to describe outcomes in comparison to the Dutch population, supplemented with disease-specific outcomes.

## 2 Methods

### 2.1 Study population and procedure

This study was a cross-sectional multicenter study in 9 Dutch Hemophilia Treatment Centers: Amsterdam University Medical Center in Amsterdam, Van Creveldkliniek in Utrecht, Erasmus University Medical Center in Rotterdam, University Medical Center in Groningen, Leiden University Medical Center in Leiden, Haga Hospital in The Hague, Hemophilia Treatment Center Nijmegen-Eindhoven-Maastricht. Data collection was conducted from September 2024 to February 2025. Patients were eligible if they were adults (aged ≥18 years), able to read and write Dutch, and diagnosed with hemophilia (men: all severities; women: clotting factor VIII/IX ≤50%) or women diagnosed with VWD (women: all types) at a Hemophilia Treatment Center. Women with VWD were additionally included because they frequently experience bleeding symptoms that overlap with those of women with hemophilia. Furthermore, women with hemophilia represent a relatively small patient population. Including women with VWD therefore enabled the inclusion of a larger cohort of women with CBDs. Patients were invited to participate in this study by their healthcare professional (HCP) or through a call on the platforms of the Dutch Hemophilia Patient Association (*Nederlandse Vereniging voor Hemofilie Patiënten; voor iedereen met een erfelijke stollingsstoornis* [NVHP]). Patients were excluded if they were diagnosed with an acquired bleeding disorder. Once patients indicated their willingness to participate, they were sent an email with a personal link to the research website (https://promis-seks.nl). Participants were asked to complete a digital informed consent form before they could proceed with the measures. After participation, participants received a €10-€15 gift card. Approval for this study was obtained from the medical ethical committee of the Amsterdam University Medical Center (W22_164 # 22.213).

### 2.2 Measures

#### 2.2.1 Patient characteristics

The sociodemographic questionnaire included items about clinical characteristics (eg, diagnosis, use of prescribed medication) and participant characteristics (eg, age, level of education, relationship status, and sexual orientation).

#### 2.2.2 PROMIS Sexual Function and Satisfaction v2.0

The PROMIS SexFS assesses multiple domains of sexual function and sexual satisfaction in both sexually active and inactive individuals. Table 1 [32] summarizes the measures included in the present study: 6 screener items and 12 PROMIS SexFS measures (6 full measures and 6 custom short forms). The selection of the 12 PROMIS SexFS measures was based on previous work [27] in which measures were selected for translation based on their relevance, a balance between function-related and satisfaction-related domains, and minimization of participant burden. Based on the screener items, sexually active participants (defined as any type of sexual activity in the past 30 days) completed the measures for sexually active individuals. Items from the Sexual Activities measure were dived into items applicable to sexually inactive and sexually active individuals, with participants completing only the relevant items. Oral and anal discomfort measures were completed only by participants who reported having experience with oral and/or anal sexual activity. This differed from the original development of the PROMIS SexFS [33], to reduce response burden, and is consistent with the questionnaire completion approach of the previous study validating the PROMIS SexFS in the Dutch general population [27]. For all measures, items queried the past 30 days, except for one item of the Sexual Function Screener (“Are you in a relationship that could involve sexual activity?”). Response options vary by item and include binary (yes/no), nominal, and ordinal (eg, 1 = not at all; 2 = a little bit; 3 = somewhat; 4 = quite a bit; 5 = very much) categories specific to each PROMIS SexFS item. The item banks were developed using the Item Response Theory (IRT) framework, although not all measures use IRT-based calibration, which allows translation of individuals’ responses into estimates of functioning, or IRT-based scoring, which allows calculation of a T-score. Of the 12 measures, 5 are IRT-calibrated and scored, 1 is IRT-calibrated and unscored, 1 is uncalibrated and scored, and 5 are uncalibrated and unscored. For IRT-calibrated measures, a T-score, which is a standardized transformation of θ, can be calculated by using the HealthMeasures Scoring Service (https://www.assessmentcenter.net/ac_scoringservice), based on item calibrations from the US general population. T-scores are expressed on a scale with a mean ± SD of 50 ± 10 in the US general population. For item banks that are uncalibrated but scored, a table can be used to transform a raw summed score into a SexFS-specific T-score. This score is a transformation of the observed average score centered by the US general population weighted mean and SD. A higher T-score represents a greater presence of the measured concept. Item banks that are both uncalibrated and unscored are intended to be used descriptively and interpreted at item-level [32].

**Table 1:** Dutch PROMIS SexFS administered in this study.

Measure name	Requires sexual activity	Number of items	Measure calibration using IRT	Item scoring	Scoring method
1. Sexual Function Screener Items	N/A	6	N/A	N/A	N/A
2. Interest in Sexual Activity^a^	No	2	No	Yes	Scoring table
3. Factors Interfering with Sexual Satisfaction (SF)^b^^,^^c^	No	17	No	No	Descriptive/item-level
4. Bother Regarding Sexual Function (SF)^b^^,^^c^	No	4	No	No	Descriptive/item-level
5. Satisfaction with Sex Life^a^	No	5	Yes	Yes	Scoring service
6. Sexual Activities^a^^,^^c^	Yes	15	No	No	Descriptive/item level
7. Orgasm-Pleasure (SF)^a^	Yes	2	Yes	Yes	Scoring service
8. Therapeutic Aids for Sexual Activity (SF)^a^^,^^c^	Yes	3	No	No	Descriptive/item-level
9. Erectile Function (men)^a^	Yes	11	Yes	Yes	Scoring service
10. Vaginal Lubrication for Sexual Activity (women)^a^	Yes	6	Yes	Yes	Scoring service
11. Vaginal Discomfort with Sexual Activity (women)^b^	Yes	11	Yes	Yes	Scoring service
12. Oral Discomfort with Sexual Activity (SF)^b^	Yes	1	Yes	No	Descriptive/item-level
13. Anal Discomfort with Sexual Activity (SF)^b^	Yes	2	No	No	Descriptive/item-level

IRT, Item Response Therapy; N/A, not applicable; SF, custom short form, see Supplementary Table 1 for included item IDs.

a Measure assessing functioning.

b Measure assessing symptoms.

c Measure is a collection of individual items, not intended to be unidimensional [32].

#### 2.2.3 Disease-specific items

Disease-specific nominal and ordinal items were formulated based on qualitative data gathered in our previous studies in individuals with CBDs [11,12]. For men, the items addressed possible limitations in sex positions, feelings of insecurity, hematospermia, bleeds during sexual activity, and the influence of hemophilia treatment on satisfaction with sex life. For women, the items focused on the impact of menstruation on sexual satisfaction, bleeds resulting from penetrative sex, and concerns about the visibility of bruises. For both men and women, disease-specific items also covered the influence of an overly cautious partner, positive or negative effects on relationships due to the CBD, (fear of) pain, fear of bleeds, and the extent to which discussing sexual function and sexual satisfaction with HCPs occurs. Open-ended items were also included to gain more insight into how these factors influence patients’ experiences. In formulating the disease-specific items and response options, the authors remained as close as possible to the structure and formulation of the PROMIS SexFS items. The disease-specific items supplement the official PROMIS items and were intended for additional descriptive use.

#### 2.2.4 Open-ended evaluation question

An open-ended evaluation question was included to collect qualitative feedback regarding the measures’ relevance and content (“*We are interested in your feedback or suggestions regarding the questionnaire. Your comments may concern any aspect, such as the content of the questions, the length, or your impression of the items*.”).

### 2.3 Statistical analyses

Before the analyses, data was screened to improve quality and reliability. Data from participants were removed if they used a specific scoring pattern (eg, no variation) or answered with the best or worst response category on all items (flatliners). No participants met these exclusion criteria. After data screening, sociodemographic characteristics of participants were summarized using descriptive statistics. Psychometric analyses were conducted to evaluate the performance of the scored PROMIS SexFS measures in adults with CBDs by testing reliability, internal consistency, known-group validity, and feasibility. No formal sample size calculation was performed; however, the sample size was considered sufficient for the planned psychometric analyses [34]. The Statistical Package for Social Sciences (SPSS) version 28.0 was used for all data analyses.

#### 2.3.1 Measurement properties – reliability, validity, and feasibility

Reliability was assessed using the SE of T-scores. Within IRT, each response pattern corresponds to a specific T-score with an associated measurement error, expressed as the SE. An SE ≤3.2 signifies a reliability of ≥90% [35,36]. This has been considered a minimum requirement for use of PROMs in individual patients [37]. We examined the percentage of scores with an SE ≤3.2 for the calibrated measures. Internal consistency was assessed using item–total correlations. Item–total correlations were calculated for each individual item to evaluate how well it corresponds with the total T-score of the scored measures; item–total correlations >0.40 were considered sufficient [38].

Known-group validity was evaluated by comparing scores between predefined subgroups based on the literature [14,[39], [40], [41], [42], [43]]. PROMIS T-scores were compared between individuals with and without a partner for Satisfaction with Sex Life (participants in a relationship were expected to report higher satisfaction), men above and below age 40 for Erectile Function (older men were expected to report worse erectile function due to aging, hemophilia treatment availability, and possible hepatitis C virus/HIV infection), and women above and below age 50 (ie, around natural menopause) for Vaginal Lubrication with Sexual Activity (older women were expected to report worse vaginal lubrication). For other measures, no hypotheses were defined due to the unsuitability of the collected data or the absence of a clear expectation.

To assess feasibility for use in clinical practice, the percentage of lowest or highest scores was calculated for the 6 calibrated and scored PROMIS SexFS measures that consisted of >1 item. High proportions of extreme scores may indicate a limited ability to distinguish between individuals, particularly when this occurs at the symptomatic end of the scale [44,45].

#### 2.3.2 Relevance

At the end of the survey, participants completed an open-ended question to evaluate the completed measures. Responses were analyzed in MAXQDA Plus 2022 (VERBI software) using inductive thematic analysis (by T.v.G.) [46] after which themes were discussed and agreed upon by 2 researchers (T.v.G. and L.T.). Responses were excluded from the thematic analysis if they were unrelated to the evaluation question (eg, personal experiences, questions about the study) or were noninformative (eg, “*no comment*,” “*nothing*,” “*x*,” dashes). Themes are reported in an order that reflects their frequency mentioned in the responses.

#### 2.3.3 Outcomes

Sexual function and sexual satisfaction in the participants with CBDs were assessed using the PROMIS SexFS. Mean T-scores for the scored PROMIS SexFS measures were calculated and compared with the reference data of the general population of the Netherlands [27], using independent samples *t*-tests. Results are reported as mean T-scores, *P* values, effect sizes (Cohen’s d), and confidence intervals. No correction for multiple comparisons was applied because the PROMIS domains were prespecified and were considered distinct outcomes. Results on measures that are both uncalibrated and unscored are reported descriptively (Table 4).

Descriptive statistics for the disease-specific items are reported. Percentages are given for the group of participants that completed the item and for whom the item was applicable. Per disease-specific theme, 1 illustrative quote from the open-ended items is presented.

## 3 Results

### 3.1 Sociodemographics

In total, 231 individuals participated in this study, of which 16 participants discontinued before completing the sexual activity screener. Based on the sexual activity screener, 17 participants (7.4%) reported no sexual activity and consequently completed only the measures that did not require sexual activity, and 198 participants (85.7%) completed the PROMIS measures designed for sexually active individuals. Some participants did not complete all PROMIS SexFS measures, resulting in varying sample sizes across measures (range 0%-9.4% per PROMIS SexFS measure).

The mean age of all participants was 44 years (range 18-81), around half were male (54%), most were married/cohabitating (63%), and 91% identified themselves as heterosexual. Of the male participants, 41% were diagnosed with mild hemophilia A. Of the female participants, 55% were diagnosed with VWD type 1 (see Table 2 for more details).

**Table 2:** Participant characteristics.

Patient characteristics			Total sample		Sexually active		Sexually inactive	
			Valid *N*	Mean ± SD	Valid *N*	Mean ± SD	Valid *N*	mean ± SD
Age, years, mean ± SD			231	44.20 ± 16.33 (range 18-81)	198	43.20 ± 15.94 (range 18-81)	17	48.78 ± 18.78 (range 20-77)
			**Valid *N***	***n* (%)**	**Valid *N***	***n* (%)**	**Valid *N***	***n* (%)**
Sex (male)			231	124 (54%)	198	109 (55%)	17	6 (35%)
Diagnosis^a^			231		198		17	
Male	Hemophilia A	Severe		42 (34%)		36 (38%)		1 (17%)
		Moderate		17 (14%)		15 (15%)		1 (17%)
		Mild		51 (41%)		44 (46%)		4 (67%)
		Unknown		1 (1%)		1 (1%)		0
	Hemophilia B	Severe		4 (3%)		4 (31%)		0
		Moderate		2 (2%)		2 (15%)		0
		Mild		7 (6%)		7 (54%)		0
Female	Hemophilia A (FVIII <50%)			15 (14%)		12 (14%)		1 (9%)
	Hemophilia B (FIX <50%)			5 (5%)		4 (5%)		0
	von Willebrand disease	type 1		59 (55%)		50 (56%)		6 (55%)
		type 2		21 (19%)		17 (19%)		3 (27%)
		type 3		6 (6%)		6 (7%)		0
		type unknown		1 (1%)		0		1 (9%)
Treatment of hemophilia (only men)^b^			124		109		6	
Prophylaxis with clotting factor concentrate				25 (20%)		23 (21%)		0
Prophylaxis with emicizumab				26 (21%)		21 (19%)		1 (17%)
On-demand treatment				73 (59%)		65 (60%)		5 (83%)
HIV infection (only men^c^)^d^			77	5 (7%)	57	5 (8%)	5	0
Past/current HCV infection (only men)^e^			96	42 (45%)	74	35 (43%)	5	4 (80%)
Methods to reduce or delay menstrual bleeding (only women)^f^			107		89		11	
Oral contraceptives/other hormonal methods				48 (45%)		41 (46%)		5 (46%)
Tranexamic acid				40 (37%)		34 (38%)		4 (36%)
DDAVP nasal spray				8 (8%)		7 (8%)		1 (9%)
**Sociodemographic characteristics**								
Educational level^g^			231		198		17	
No education				2 (1%)		1 (1%)		1 (6%)
Primary				3 (1%)		2 (1%)		2 (12%)
Secondary				108 (47%)		93 (46%)		7 (41%)
Tertiary				117 (51%)		101 (51%)		7 (41%)
Other				1 (0%)		1 (1%)		0
Current relationship status			231		198		17	
Married/cohabiting				145 (63%)		128 (65%)		7 (41%)
Divorced				14 (6%)		9 (4%)		3 (18%)
Widowed				5 (2%)		5 (3%)		0
Single				37 (16%)		29 (15%)		4 (23%)
Other^h^				27 (12%)		24 (12%)		3 (18%)
Prefer not to say				3 (1%)		3 (2%)		0
Sexual orientation			231		198		17	
Heterosexual				211 (91%)		181 (91%)		15 (88%)
Homosexual				8 (4%)		7 (4%)		0
Bisexual				6 (3%)		5 (3%)		1 (6%)
Pansexual				3 (1%)		2 (1%)		1 (6%)
Asexual				1 (0%)		1 (1%)		0
Other^i^				1 (0%)		1 (1%)		0
Prefer not to say				1 (0%)		1 (1%)		0

Percentages are calculated based on the number of participants with available data for each variable (valid *N*).

DDAVP, 1-8-deamino-d-arginine vasopressin; FIX, factor IX; FVIII, factor VIII; HCV, hepatitis C virus; ISCED International Standard Classification of Education.

a Percentages are given within each sex (male/female) group.

b Only participants who identified as male were presented with this question. Percentages are presented based on this group.

c HIV status was assessed only in men with hemophilia, as none of the participating women had a known history of receiving plasma-derived clotting factor concentrates.

d Only participants who identified as male and were born before 1985 were presented with this question. Percentages are presented based on this group.

e Only participants who identified as male and were born before 1992 were presented with this question. Percentages are presented based on this group.

f Only participants who identified as female were presented with this question. Percentages are presented based on this group.

g Participants’ highest completed level of education. Education level was categorized according to ISCED levels: Primary education (ISCED level 1), Secondary education (ISCED levels 2 and 3), Tertiary education (ISCED levels 6 and 7).

h Mostly long-distance relationships.

i Specified as “it is not very clear.”

### 3.2 Measurement properties—reliability, validity, and feasibility

For all measures, >78% of participant scores met the minimum requirement for reliability (SE ≤3.2) [47,48]. In addition, item–total correlations were >0.43 for all items within these measures (see Table 3).

**Table 3:** Reliability, lowest and highest scores, and mean scores of the PROMIS SexFS measures.

PROMIS SexFS measure	N	Reliability (%)	Range item–total correlation	Lowest score (%)	Highest score (%)	T-score, M (SD)
Interest in Sexual Activity^a^^,^^b^^,^^c^	231	N/A^d^	0.95-0.97 (M = 0.96)	2.6	1.7	47.97 (8.88)
Satisfaction with Sex Life^a^^,^^b^	198	94.5	0.74-0.89 (M = 0.84)	1.5	4.5	49.67 (6.80)
Orgasm-Pleasure^a^^,^^b^^,^^c^	198	78.2	0.92-0.95 (M = 0.93)	0	25.4	49.05 (7.73)
Erectile Function^a^^,^^b^	106	79.9	0.43-0.82 (M = 0.72)	0.9	17	48.83 (8.21)
Vaginal Lubrication for Sexual Activity^a^^,^^b^	87	84.4	0.77-0.87 (M = 0.81)	0	16.1	50.66 (8.44)
Vaginal Discomfort with Sexual Activity	87	91.8	0.49-0.89 (M = 0.78)	9.2	0	54.63 (7.94)

US cutoff T-scores: <30 → Very low; 30-39 → Low; 40-59 → Average; 60-69 → High; ≥70 → Very high.

M, mean.

a Measure assessing functioning.

b Both items of the measure had to be completed and no “not applicable” (eg, “Have not had an orgasm/climax in the past 30 days”) answers can be given to calculate a T-score.

c No standard errors are generated for this measure, as this measure is not calibrated.

d Measure assessing symptom.

Two hypotheses regarding expected differences between subgroups on specific measures were confirmed. Participants in a relationship reported significantly higher Satisfaction with Sex Life than participants who were currently single (50.19 vs 47.70; *P* = .02, d = 0.37, 95% CI 0.02-0.71). In addition, men older than 40 reported significantly more difficulty with Erectile Function than men younger than 40 years (46.7 vs 51.8; *P* < .001, d = 0.66, 95% CI 0.26-1.05). Too few female participants aged >50 years participated in the current study to conduct analyses comparing age subgroups on Vaginal Lubrication. For the measures where no hypotheses were defined, no statistical analyses were performed.

Highest scores were found within a range of 0 to 25.4%, where the percentages were the highest specifically for the measures Vaginal Lubrication for Sexual Activity, Erectile Function, and Orgasm-Pleasure (range 16.1%-25.4%; Table 3). Lowest scores were found within a range of 0 to 9.2%.

### 3.3 Relevance

Of the 215 participants who completed the open-ended evaluation question, the responses of 151 participants could be included in thematic analyses. **Overall feedback was positive**, with participants describing the survey as “*okay*,” “ *clear*,” and “*included all important themes*.” Some participants noted that items appeared **similar or overlapping**. Another aspect mentioned was a lack of **opportunities to explain certain answers**. One participant said: “*My partner has erectile dysfunction. Therefore, we do not have much penetrative sex, but we do have a good sex life. This is not reflected well in the way the questions are asked*.” Another participant commented: “*It could be helpful to give people the opportunity to explain why they think they feel tired or stressed* [which affects satisfaction with sex life].” Furthermore, the **quality of item wording and formulation** of the items was mentioned both positively and negatively. Responses included “*Well-formulated questions*” and “*The questions sometimes confused me because they could be interpreted in different ways*.” Another aspect mentioned was having to complete **questions that were not applicable**: “*I have been single for years and don’t have a partner. Many questions could have been skipped by first asking if someone is sexually active*” and “*When you indicate that you have not had a sexual partner in the past few days, you would prefer not to receive twenty follow-up questions about what you did with your partner*.” Some participants felt the topic was a bit **too intimate**. Furthermore, some noted that the **recall period** was too narrow and captured only momentary experiences. For example: “*I seriously wonder whether the 30-day period that is often used is long enough to get an adequate average picture of the participant’s situation*.” **Disease-specific** remarks were also made, such as the importance of knowing when a patient was diagnosed or that the topic felt unrelated to a bleeding disorder. Opinions on the **length of the questionnaire** varied. Participants also mentioned missing a “*not applicable*” **response option** for some items. Some female participants noted a need for more questions about **menstruation**, as this is an important topic in the context of bleeding disorders and sometimes even more a taboo than sexual function and sexual satisfaction. Lastly, a homosexual participant mentioned the questionnaire was not **inclusive/sensitive to sexual orientation**: “*As a homosexual man, I find the questions about vaginal penetration unnecessary*.”

### 3.4 Outcomes

In comparison with Dutch reference data, women with CBDs reported more Vaginal Discomfort with Sexual Activity (54.6 vs 50.6; *P* < .001, d = 0.48, 95% CI 0.25-0.70), men with CBDs reported better Erectile Function (48.8 vs 46.7; *P* = .008, d = 0.26, 95% CI 0.05-0.47), and the total group of individuals with CBDs reported more Pleasure with their Orgasms (49.1 vs. 47.1; *P* = .002, d = 0.23, 95% CI 0.08-0.39) and more interest in sex life (48.0 vs. 46.1; *P* = .005, d = 0.19, 95% CI 0.05-0.32). No statistically significant differences were found for the other measures (see the Figure for T-scores and Table 4 for descriptive statistics of the uncalibrated and unscored item banks).

**Figure:**
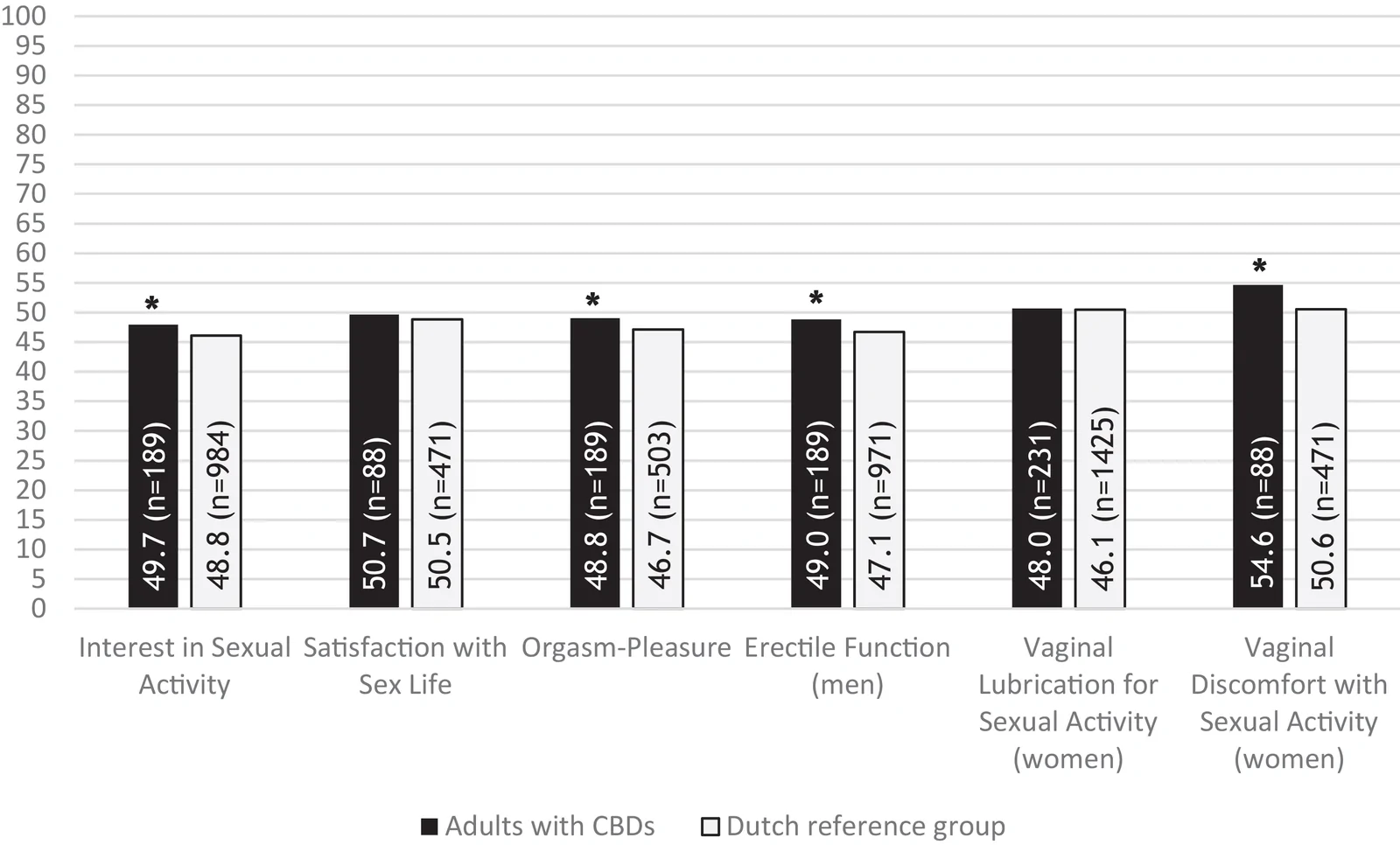
Mean T-scores for adults with congenital bleeding disorders and the Dutch reference group [27]. Higher T-scores indicate higher levels of the measured concept. CBD, congenital bleeding disorder. ∗*P* < .05.

**Table 4:** Descriptive statistics of the uncalibrated PROMIS SexFS measures.

PROMIS SexFS measure	*N*	Descriptive statistics^a^
Factors Interfering with Sexual Satisfaction	226	Of the 17 items, participants most often indicated fatigue, pain, moving the body, stress, and feeling unattractive as interfering factors. Fatigue was the most affecting factor, with 25% of the participants reporting impact.
Bother Regarding Sexual Function		
Male	120	28% of the male participants reported being bothered by their level of interest in sexual activity, 20% by their ability to have an erection, and 15% by their ability to have an orgasm.
Female	103	42% of the female participants reported being bothered by their level of interest in sexual activity, 26% by the ability to have an orgasm, and 57% by discomfort or pain inside the vagina.
Sexual Activities		
All	216	40% of participants reported masturbation while with a partner being present and 60% reported masturbation while alone. Around 80% of participants engaged at least once in a sexual activity in the past 30 days (eg, hugging, kissing).
Male (sexually active)	107	27% of male participants reported masturbation while with a partner being present, 78% reported masturbation while alone, and 65% reported penetrative sex.
Female (sexually active)	87	30% of female participants reported masturbation while with a partner, 55% reported masturbation while alone, and 84% reported engaging in penetrative sex.
Therapeutic Aids for Sexual Activity		
Male	107	51% of male participants reported lubricant use and 10% reported use of performance enhancing pills (eg, Viagra, Cialis, Levitra).
Female	87	46% of female participants reported lubricant use.
Oral Discomfort with Sexual Activity^b^	109	15% of participants who reported experience with oral sexual activity experienced discomfort.
Anal Discomfort with Sexual Activity^b^	16	37% of participants who reported experience with anal sexual activity, bled from their anus/rectum, and 38% experienced anal discomfort during sexual activity.

a Assessed over the past 30 days.

b Measure assessing symptom.

For the disease-specific items, the descriptive statistics are presented in Table 5.

**Table 5:** Overview of disease-specific themes with descriptive and qualitative results.

Disease-specific factors	*N*	Descriptive results^a^		Illustrative quotes (open-ended items)
**All participants**				
Overly cautious partner	224	27% of participants reported that their partner is cautious during sexual activity. Of these, 48% reported that this affected their satisfaction with sex life.		*“Less enjoyment (for both), because you cannot fully let go to do what you want.” Male, 34 years, severe CBD*
Effect on relationship	224	10% of participants reported a negative effect on their relationship, 1% reported a positive effect.		*“When I bump into something, it causes me stress, and my partner is also affected by this; my mood worsens directly with the stress.” Male, 44 years, mild CBD*
Pain	163	58% of participants reported that pain affected their satisfaction with sex life.		*“Because of the frequent pain, I have lost trust in my body. At the moment, it is simply difficult to enjoy my sex life.” Female, 58 years, severe CBD*
Fear of pain	129	48% of participants reported that the fear of pain affected their satisfaction with sex life.		*“I am afraid that I would experience pain during sex and might start bleeding.” Female, 19 years, severe CBD*
Fear of bleeds	117	42% of participants reported that the fear of bleeds affected their satisfaction with sex life.		*“I am not as physically active as I would like to be because I am scared* [to have a bleed].*” Male, 31 years, severe CBD*
Discussing sexual function and sexual satisfaction with HCP	210	24% of participants had ever discussed sexual function and sexual satisfaction. Discussion were mainly with physician at HTC/hospital (20%)		*“I cannot recall it specifically, but it* [sexual function and sexual satisfaction] *was discussed at some point when I was younger.” Male, 35 years, severe CBD*
		Satisfaction with discussions (1–10 scale):		
		8.0	Median	
		7.8	Mean	
		Preferred HCP to discuss with:		
		40%	Physician	
		32%	Nurse	
		21%	General practitioner	
		21%	Female HCP (63% female respondents)	
		20%	Member of regular treatment team	
		27%	Do not wish to discuss this topic	
**Male**				
Limitations in moving body	73	57% of participants reported that limited body movement affected their satisfaction with sex life.		*“Certain sexual positions are not possible due to physical limitations.” Male, 41 years, severe CBD*
Insecurity	71	38% of participants reported that insecurity affected their satisfaction with sex life.		*“When insecurities are present, sexual performance becomes more difficult.” Male, 50 years, mild CBD*
Hematospermia	121	21% of participants experienced hematospermia at least once a year. Of these, 53% reported that this affected their satisfaction with sex life.		*“I felt ashamed toward my sexual partner, and it is also a moment of shock for me each time.” Male, 53 years, severe CBD*
Bleeds	121	12% of participants experienced a bleed during or after sexual activity. Of these, 21% reported that this affected their satisfaction with sex life.		*“*[Satisfaction with sex life is affected by] *having my girlfriend to stop certain actions or repeatedly warning her to be careful.” Male, 34 years, severe CBD*
Influence of hemophilia specific medication	121	39% of participants used hemophilia specific medication. Of these, 5% reported that this affected their satisfaction sex life.		*“I feel more secure sexually with Hemlibra. I do not need to think about it or worry beforehand.” Male, 30 years, severe CBD*
Traumatic memories to treatment	41	7% of participants reported that traumatic memories affected their satisfaction with sex life.		*“Because of my history with HIV and AIDS, which occurred right during my adolescence, sex became associated with death and life-threatening danger. HIV was a reason for exclusion, avoidance, and discrimination. My body is marked by AIDS and by the first medications used to treat it.” Male, 53 years, severe CBD*
**Female**				
Menstruation	84	67% of participants reported that menstruation affected their satisfaction with sex life.		*“A lot of cramping before and during menstruation, as well as heavy bleeding, causes no sexual interest during that time.” Female, 25 years, mild CBD*
Bleeds	56	36% of participants experienced a bleed during or after sexual activity. Of these, 56% reported that this affected their satisfaction sex life.		*“It also causes a sense of shame. My friends can have rough sex without bleeding afterward*.*” Female, 21 years, severe CBD*
Bruises	81	22% of participants reported that bruises affected their satisfaction with sex life.		*“People have questions about it, assume you are being abused, or think you are very fragile. Besides that, it does not look attractive.” Female, 31 years, severe CBD*

CBD, congenital bleeding disorder; HTC, hemophilia treatment Center; HCP, healthcare professional.

a Assessed over the past 30 days, unless specified otherwise.

## 4 Discussion

This study evaluated the psychometric properties and suitability of the PROMIS SexFS for use in Dutch adults with CBDs. As expected, the PROMIS SexFS measures demonstrated sufficient reliability for the scored measures in this study population. Evidence of known-group validity was observed for the selected calibrated measures in which this could be assessed. Highest scores were found for 3 measures that assess functioning. These findings suggest that many participants experience high levels of sexual functioning and satisfaction and may indicate that the measures are less sensitive to differences at the positive end of the scale [44]. However, this has limited negative consequences for clinical care, as it does not prevent the identification of patients who may need support for challenges in sexual function and sexual satisfaction.

Participants evaluated the relevance of the PROMIS SexFS generally positive, but noted some limitations, such as items being not applicable to them. This issue could be reduced by using more screener items. For example, in addition to the screener asking about any sexual activity in the previous 30 days, a screener for sexual activity with a partner could prevent irrelevant items for participants not sexually active with a partner. However, we chose not to use this second screener because many measures also assess solo sexual activity, and some items refer to both unpartnered and partnered sexual activity. The findings underscore the importance of carefully selecting screener items, using items that are appropriate for the patient, and clearly defining sexual activity (ie, including both solo-sex and sex with a partner). Participants also noted that they missed the possibility to clarify their answers. Because the PROMIS SexFS was used solely for research purposes and responses were not discussed with participants, opportunities to clarify responses were absent. Discussing responses in clinical practice may address this concern, consistent with previous research demonstrating the added value of discussing PROMs in clinical care [49,50]. Furthermore, the PROMIS SexFS was perceived as not being inclusive and mostly heteronormative by one participant. This limitation was also noted by the researchers during the translation process of the PROMIS SexFS in a previous study [27]. Unfortunately, PROMIS translation guidelines prevented modifications to the content that could have increased inclusivity.

Comparing the mean T-scores of individuals with CBDs with the Dutch reference group [27] showed that women with CBDs reported more vaginal discomfort with sexual activity than women from the general population. This may be explained by the possibility of bleeding during sexual activity. Men with hemophilia reported better erectile function than the Dutch reference group, while use of therapeutic aids for enhancing erectile function was comparable between both groups. Moreover, the whole sample reported more orgasm pleasure and more interest in sexual activity than the Dutch reference group. Possible explanations for these differences were not examined in this study. It is important to emphasize that although significant, identified differences were small to moderate (with the highest effect size d = 0.48). Further research is needed to explain the differences between individuals with CBDs and the reference group. In the other domains, no statistical differences were found between individuals with CBDs and individuals from the general population.

Generic measures, consisting of standardized items such as those of the PROMIS SexFS, allow for comparison across various populations. However, using additional disease-specific items can capture outcomes that might otherwise be overlooked [51], and combining both provides a more comprehensive assessment. In the present study, disease-specific items showed the possible role of disease-specific factors (eg, bleeds, bruises) affecting satisfaction with sex life. Factors that affected sexual satisfaction the most were a partner being overly cautious during sexual activity, (fear of) pain, (fear of) bleeds, limitations in moving the body (specifically in men), and (heavy) menstruation in women. These findings are consistent with our previous qualitative studies that found men with hemophilia felt restricted by their joint movements and reported partners sometimes being overly cautious. Bleeding during or after sexual activity was reported in both men (eg, hematospermia, iliopsoas muscle bleed) [11] and women (eg, vaginal bleeding). Additionally, women with CBDs reported feelings of insecurity or shame due to heavy menstruation, often leading to refraining from sexual activity during menstruation [12].

The majority of participants in the present study reported that sexual function and sexual satisfaction were not discussed with HCPs, while over 75% of participants indicated that they do want to discuss sexual function and sexual satisfaction. These findings are consistent with results from a nationwide study [17] and previous qualitative studies [11,12]. When discussions about sexual function and sexual satisfaction occurred, they were rated with a mean score of 8. Thus, despite being sensitive, discussions about the topic of sexual function and sexual satisfaction are received positively. These findings underscore the importance of discussing this topic, or asking permission to do so, given the potential difficulties this group may experience.

Taken together, the findings of this study support the combined use of generic and disease-specific items for comprehensive assessment and facilitation of conversations about sexual function and sexual satisfaction. It also underscores the need for informational materials to support patients, raise awareness, and facilitate conversations.

Strengths of this study include its multicenter design and the inclusion of women with CBDs, as data within this group are limited [52]. However, a limitation is that treatment characteristics were not collected for women with CBDs, which may limit interpretation and comparability. Additionally, selection bias may be present, as participating individuals with CBDs may represent individuals who are more engaged and more comfortable completing items regarding sexual function and sexual satisfaction. Further limitations that should be considered include several aspects of the PROMIS SexFS. Although the PROMIS SexFS aims to be inclusive for individuals with diverse sexual orientations, participant evaluations highlighted the heteronormative wording of some items. Furthermore, response options are coded differently across PROMIS SexFS measures. For example, the response option “have not done/tried in the past 30 days,” is treated as missing in some measures but assigned a score in others. This may influence score interpretation and should be taken into account when interpreting results. When using these measures in clinical practice, discussing patients’ answers using literal responses instead of T-scores may be preferable. Accordingly, previous research has shown that literal response categories carry important clinical meaning and are valued by clinicians and patients who use PROMs in daily practice [50,53], (Groenewegen A, personal communication). A further consideration is that hypotheses for known-group validity were not defined for every scored measure. When hypotheses were formulated, only one hypothesis per measure was specified and tested. Methodological guidelines recommend testing multiple hypotheses per measure and confirming the majority of predefined hypotheses [34,54]. However, given the influence of multiple factors on sexual function [55] and the available data, no additional hypotheses were specified or tested. Therefore, known-group validity should be interpreted with caution.

Recommendations for future research include exploring sexual function and sexual satisfaction in larger populations with CBDs, which would allow a better understanding of factors influencing sexual functioning and sexual satisfaction and more detailed analyses of subgroups (eg, elderly patients, specific CBDs, gender and sexual diversity). Future studies should also explore the use of computer adaptive testing (CAT) for calibrated measures. In CAT (a computer-administered test), item presentation is determined by the patient’s response to previous ones. After each item, the patient’s score is estimated, and item administration continues until a certain reliability is reached. Administering PROMIS measures using CAT can reduce patient burden while maintaining reliability [56,57]. Furthermore, future work should focus on the adequate implementation of the PROMIS SexFS in clinical practice. This could involve using specific domains of the PROMIS SexFS to monitor sexual function and sexual satisfaction and to facilitate conversations about these topics. Developing tools for HCPs and informational materials based on insights from patients, the PROMIS SexFS, and open-ended disease-specific items could help facilitate conversations and improve information provision with patients.

The results of this study suggest that the Dutch versions of the PROMIS SexFS measures are suitable for assessing sexual function and sexual satisfaction in individuals with CBDs in research and clinical practice. The PROMIS SexSF indicated higher levels of Vaginal Discomfort with Sexual Activity among women with CBDs and higher levels of Erectile Function among men with hemophilia, compared with the general Dutch population. In the whole sample, higher levels of Orgasm-Pleasure and Interest in Sexual Activity were observed. Given the length of the full PROMIS SexFS, using appropriate screener items and short forms or CATs are important to reduce patient burden [56,57] in both research and clinical practice. The addition of (open-ended) disease-specific items to the generic PROMIS SexFS provides a comprehensive assessment and, together with findings from the PROMIS SexFS, can support conversations about sexual function and sexual satisfaction in clinical practice.
