# Supplementary material for: Psychometric properties and outcomes of the PROMIS Sexual Function and Satisfaction measures in individuals with congenital bleeding disorders
Source: Res Pract Thromb Haemost. 2026 Sep 2;10(6):106937. doi: 10.1016/j.rpth.2026.106937 (PMC13633915; doi:10.1016/j.rpth.2026.106937)
Supplement: Supplementary file 1 [file mmc1.docx]

**Supplementary material**

**Supplementary Table 1. Item IDs of custom short forms**

| Measure Name | Items IDs |
| --- | --- |
| Factors Interfering with Sexual Satisfaction (SF) | SFFAC101  SFFAC102r  SFFAC103r  SFFAC202  SFFAC203  SFFAC204  SFFAC205  SFFAC207  SFFAC208  SFFAC209  SFFAC210  SFFAC212  SFFAC214  SFFAC215  SFFAC216  SFFAC219  SFFAC220 |
| Bother Regarding Sexual Function (SF) | SFBOT202 (female)  SFBOT204 (male)  SFBOT205  SFBOT206 |
| Orgasm-Pleasure (SF) | SFOGP201  SFOGP203 |
| Therapeutic Aids for Sexual Activity (SF) | SFAID101r  SFAID105 (male) |
| Oral Discomfort with Sexual Activity | SFODS201 |
| Anal Discomfort with Sexual Activity | SFANA104r  SFANA102r |

SF: custom short-form
